# Quantitative Assessment of Upper Limb Ataxia Using a Virtual Reality‐Based Evaluation System

**DOI:** 10.1002/acn3.70215

**Published:** 2025-10-02

**Authors:** Masayuki Sato, Takayuki Abe, Sho Aoki, Setsuki Tsukagoshi, Yasushi Yuminaka, Yoshio Ikeda

**Affiliations:** ^1^ Department of Neurology Gunma University Graduate School of Medicine Maebashi Japan; ^2^ Division of Electronics and Informatics Gunma University Graduate School of Science and Technology Kiryu Japan

**Keywords:** digital biomarker, finger‐to‐nose test, quantitative assessment, upper limb ataxia, virtual reality

## Abstract

**Objective:**

Cerebellar ataxia impairs coordination and balance, reducing quality of life. Conventional clinical scales, including the Scale for the Assessment and Rating of Ataxia (SARA) and the International Cooperative Ataxia Rating Scale (ICARS), are widely used to assess ataxia but are limited by subjectivity and inter‐rater variability. Therefore, we aimed to develop a virtual reality‐based system to objectively and quantitatively assess upper limb ataxia.

**Methods:**

A “virtual nose‐finger test” was implemented using a head‐mounted display, in which participants performed repetitive reaching tasks. Six parameters were measured: four spatial (subtracted length, trajectory ratio, terminal trajectory length, and maximum overshoot distance) and two temporal (required time and movement speed). These parameters were compared across groups, correlated with clinical scales, and analyzed for diagnostic accuracy using receiver operating characteristic curves. Motor adaptation was assessed using parameter changes across trials.

**Results:**

Ninety‐five participants were recruited: 39 with cerebellar ataxia, 30 controls, and 26 with Parkinsonian disorders. Participants with ataxia exhibited significantly greater spatial deviations and temporal variability than other groups did. Trajectory ratio, required time, and movement speed variability coefficient significantly correlated with clinical ataxia scores. The system demonstrated high diagnostic accuracy from the receiver operating characteristic analyses, and participants with ataxia showed different motor adaptations by compensating for spatial errors through reduced movement speed.

**Interpretation:**

This virtual reality‐based system enables objective, quantitative, portable, and ambulatory‐independent evaluation of upper limb ataxia, enhancing its feasibility in clinical and research settings and its potential as a biomarker for cerebellar ataxia.

## 1 Introduction

Cerebellar ataxia results in impaired coordination of limb and trunk movements, loss of balance, and slurred speech. These symptoms restrict daily activities and increase the risk of falls, reducing patients' quality of life [1]. Cerebellar ataxia has various etiologies, such as spinocerebellar ataxias (SCAs), which are primarily inherited and progressive neurodegenerative diseases, and multiple system atrophy (MSA), which is mainly sporadic and accompanied by Parkinsonism or autonomic disturbance. Despite numerous clinical trials conducted in patients with SCA or MSA, no effective therapies have been approved [2]. Notably, several problems have been indicated in the clinical trials for SCAs.

The Scale for the Assessment and Rating of Ataxia (SARA) and the International Cooperative Ataxia Rating Scale (ICARS) are the most widely used clinical scales for assessing cerebellar ataxia severity as an outcome in clinical trials [3, 4]. These clinical scales have been validated for SCAs and other diseases that cause cerebellar ataxia and have been introduced in various clinical trials, natural history studies, and clinical settings [5, 6, 7, 8]. However, as these clinical scales are based on semiquantitative assessment, they are subjected to inherent subjectivity and inter‐rater variability [9, 10]. Their reliability may also vary depending on the cerebellar ataxia severity [11]. Furthermore, because most SCAs progress very slowly, a large number of patients with SCAs are required to detect therapeutic efficacy [12, 13]. However, the prevalence of SCAs is relatively low and differs from region to region [14, 15]. These situations make it difficult to recruit enough patients with SCAs to conduct a clinical trial. These limitations highlight the need for quantitative and accurate evaluation methods, especially in clinical trials with a limited number of participants.

Thus far, various tools have been used to assess the severity of cerebellar ataxia objectively. The optoelectronic systems with digital cameras and passive markers identified poor movement smoothness in the upper limb of patients with cerebellar ataxia [16]. Another study in which a robotic device was used reported reduced movement accuracy and impaired smoothness of the upper limb in patients with cerebellar ataxia [17]. These results correlated well with the SARA or ICARS scores. However, these systems require highly specialized devices and techniques, which limit their feasibility in clinical settings. We previously reported a device with an infrared depth sensor system that can quantitatively assess gait abnormalities in patients with cerebellar ataxia and Parkinson's disease [18]. It is highly portable and affordable and can be used to gauge the severity of cerebellar ataxia and Parkinsonism, but it is obviously unsuitable for evaluating nonambulatory patients.

Recent progress in virtual reality (VR) technology has improved measurement accuracy, and VR devices are increasingly used to rehabilitate patients with cerebellar ataxia [19, 20, 21]. However, to our knowledge, only a few studies have explored VR devices for the quantitative assessment of cerebellar ataxia [22, 23]. In this study, we aimed to develop a quantitative and objective system for evaluating the severity of cerebellar ataxia using a highly accurate and compact VR device that can be used for bedside assessment.

## 2 Patients and Methods

### 2.1 Patients

The study participants were recruited from consecutive patients with cerebellar ataxia who visited the Department of Neurology at Gunma University Hospital between May 2023 and October 2024. Patients without upper limb impairment were included as control participants, and those with Parkinsonism were included as disease control participants. MSA, progressive supranuclear palsy (PSP), and Parkinson's disease (PD) were diagnosed according to established diagnostic criteria [24, 25, 26]. The diagnoses of genetic diseases, such as SCA types 6 (SCA6) and 31 (SCA31), were confirmed via genetic testing. We excluded patients with severe cognitive impairment who had difficulty understanding the evaluation task or those with visual impairment who had difficulty looking at the target. All participants provided written informed consent. The Ethics Committee of the Gunma University Graduate School of Medicine approved this study (approval number: IRB2022‐059; approval date: January 26, 2023).

### 2.2 Experimental Device and Evaluation Procedure

We developed an evaluation system using a head‐mounted display (HMD) (Oculus Quest 2, Meta Platforms Inc., USA) to measure the three‐dimensional coordinates of a fingertip and quantitatively assess upper limb movement. The system incorporated a “virtual nose‐finger test” based on the conventional nose‐finger test included in the items of the SARA and ICARS [3, 4]. The nose‐finger test is routinely used in clinical settings to evaluate the severity of upper limb ataxia and is considered an easy task for patients to perform. The evaluation program was developed using the Unity game engine (Unity Technologies, San Francisco, CA, USA). The HMD's internal camera recorded fingertip movements at 60 frames per second, enabling accurate hand tracking in a VR environment. This system allows precise detection of hand movements, and its tracking performance has an accuracy comparable with that of conventional motion capture systems [27, 28].

**FIGURE 1:**
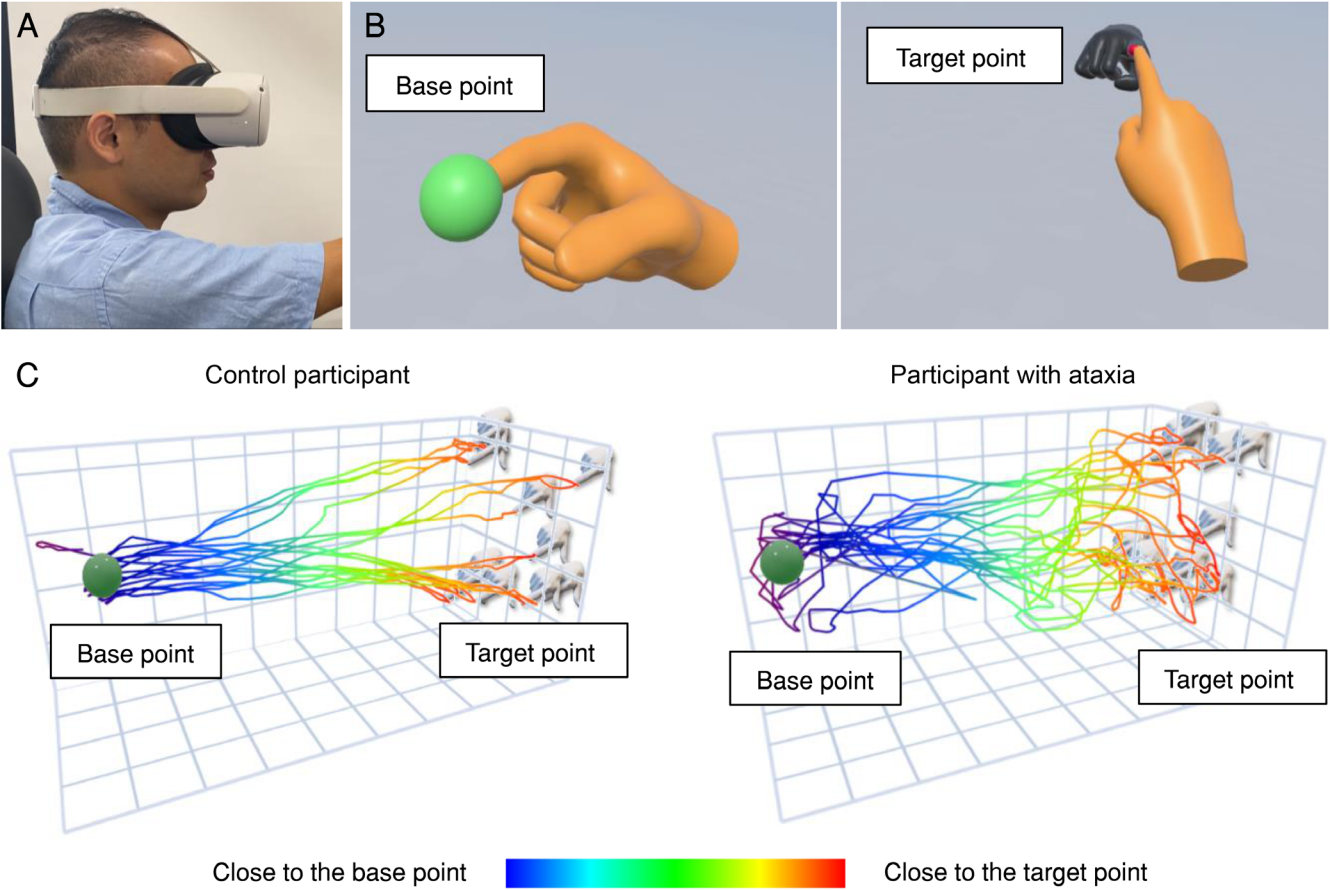
Experimental setup of the virtual reality (VR) device and the evaluation task. (A) Appearance while wearing a head‐mounted display. (B) The green sphere, which represented the base point, and the virtual hand with the red index fingertip, which represented the target point, appeared alternately in the VR space. Each participant touched these points alternately with their virtual index finger. (C) Perspective view of movement paths in one control participant and one participant with ataxia, evaluated with their dominant hand.

Before starting the VR‐based evaluation, the participants were seated and fitted with the VR device (Figure 1A). They were instructed to extend their right or left arm forward so that the positions of the target point (represented as a virtual hand with a red index fingertip) could be adjusted. Arm (left or right) lengths were measured using the VR device, and the target points were programmed to appear at a distance of 90% of the participant's arm length. During evaluation, participants were requested to alternately touch the base point appearing as a green sphere on the near side and the target point appearing as the index finger of the virtual hand on the far side (Figure 1B). The base point remained fixed, while the target point appeared at random positions within a constant distance. Once the participant touched the base or target points, each point immediately disappeared with a drum sound for feedback. Participants were instructed to perform the evaluation task as accurately as possible. Each participant repeated this task 10 times with their right hand and then with their left hand. All repetitions were counted as one trial. Each participant was requested to repeat three trials in total, and they were given ample practice time (1–2 min) before the evaluation. Representative trajectories of one control participant and one participant with ataxia, performed using their dominant hand in one trial, are shown (Figure 1C).

### 2.3 Parameters Measured Using the Virtual Reality Device

**FIGURE 2:**
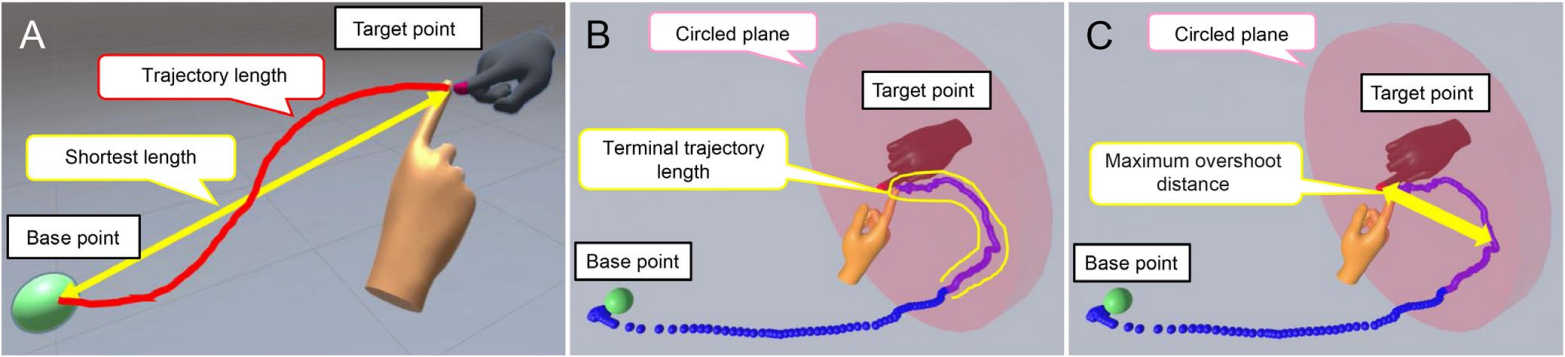
Conceptual diagrams of parameters measured using the virtual reality device. (A) The straight line connecting the base and the target points was defined as the shortest length (yellow two‐headed arrow). The red curve expressed the trajectory length of the participant's index fingertip. (B) The circled plane with a diameter of 60 cm was placed at the center of the target point. The sequential trajectory of the participant's index finger was expressed by a series of blue points. This circled plane and these blue points were invisible to the participants. The terminal trajectory length was represented as the range enclosed by the yellow line. (C) The yellow two‐headed arrow showed the length between the target point and the farthest point passing through the circled plane.

The virtual nose‐finger test measured six parameters. “Subtracted length” was calculated as the difference between the actual trajectory length and the shortest possible length, and “trajectory ratio” was calculated as the ratio of the actual trajectory length to the shortest possible length (Figure 2A). These parameters reflect the grades of decomposition and dysmetria, which are characteristic signs of cerebellar ataxia.

“Terminal trajectory length” was defined as the trajectory length traveled by the participant's index finger from the point where it crossed the circled plane to the target point (Figure 2B). “Maximum overshoot distance” was defined as the maximum distance between the target point and any point along the finger trajectory after crossing the circled plane (Figure 2C). These two parameters were specifically introduced to assess overshoot in three‐dimensional space, a hallmark of cerebellar ataxia.

Conventional two‐dimensional systems have a limited ability to evaluate anterior–posterior overshoot, which is often prominent in hypermetria. The terminal trajectory length reflects corrective feedback‐guided movement required to reach the target point, while the maximum overshoot distance reflects the maximum extent of overshoot. We also measured the average time required and speed for each one‐way movement of the participant's index fingertip as “required time” and “movement speed,” respectively. The average values of the parameters for the right and left hands were used for the analyses. In addition, the coefficients of variation (CVs) for these parameters were calculated.

### 2.4 Assessment of Motor Symptom Severity in Participants With Ataxia and Parkinsonism

Cerebellar ataxia severity was assessed using the SARA and ICARS. The SARA and ICARS total scores ranged from 0 to 40 and 0 to 100 points, respectively, with higher scores indicating greater severity. In addition, we evaluated the severity of upper limb ataxia using the SARA and ICARS upper limb sub‐scores. The SARA upper limb sub‐score comprised “finger chase,” “nose‐finger test,” and “fast alternating hand movements,” ranging from 0 to 12 points, while the ICARS upper limb sub‐score comprised “finger‐to‐nose test: decomposition and dysmetria,” “finger‐to‐nose test: intention tremor of the finger,” “finger‐finger test,” “pronation‐supination alternating movements,” and “drawing of the Archimedes' spiral on a pre‐drawn pattern,” ranging from 0 to 36 points. Furthermore, Parkinsonism severity was assessed using disease‐specific rating scales: The Unified Parkinson's Disease Rating Scale (UPDRS) for patients with PD, the Progressive Supranuclear Palsy Rating Scale (PSPRS) for patients with PSP, and the Unified Multiple System Atrophy Rating Scale (UMSARS) for MSA with predominant Parkinsonism (MSA‐P) [29, 30, 31].

### 2.5 Statistical Analyses

Statistical analyses were performed using the Statistical Package for Social Sciences Statistics version 28.0 (IBM Japan, Tokyo, Japan). All data are presented as mean ± standard deviation. Data normality was determined using the Kolmogorov–Smirnov test. Normally and non‐normally distributed data were compared among the three groups using a one‐way analysis of variance and the Kruskal–Wallis test, respectively. The Bonferroni correction was used to make multiple comparisons among the three groups. For two‐group comparisons, the chi‐square test was used for categorical data, Student's *t*‐test was used for normally distributed data, and the Mann–Whitney *U* test was used for non‐normally distributed data. Receiver operating characteristic (ROC) curves were used to evaluate the accuracy with which the equipment distinguishes participants with ataxia from the controls for each parameter measured using the VR device. Correlation analyses between each parameter and the SARA and ICARS total scores and upper limb sub‐scores in patients with ataxia were performed using Spearman's rank correlation coefficients. Multiple comparisons were performed between the first, second, and third trials of the three measurement parameters (trajectory ratio, required time, and movement speed) in each group using Friedman's test and Bonferroni correction. A *p*‐value of < 0.05 was considered statistically significant.

## 3 Results

### 3.1 Characteristics of Participants

Overall, 95 participants were included: 39 with cerebellar ataxia, 30 controls, and 26 with Parkinsonism. Of the 39 participants with ataxia, 11 had MSA with predominant cerebellar ataxia (MSA‐C) and 10 had SCA6, representing the largest subgroups. Other etiologies included one case each of SCA1, cerebellar ataxia with neuropathy and vestibular areflexia syndrome, Sjögren's syndrome, Miller Fisher syndrome, and tumefactive demyelinating lesions, as well as two cases of SCA31. The remaining 11 participants had unknown etiologies. Of the 26 participants with Parkinsonism, 18 had PD, five had PSP, and three had MSA‐P. Demographic characteristics for the control, ataxia, and Parkinsonism groups are shown (Table 1). The age at examination differed significantly between participants with Parkinsonism and the controls or those with ataxia. However, no significant difference was observed between the ages of the control participants and those with ataxia (Figure S1). No significant sex bias was observed among the groups.

**TABLE 1:** Clinical characteristics of participants in the control, ataxia, and Parkinsonism groups.

	Controls (*n* = 30)	Patients with ataxia (*n* = 39)	Patients with Parkinsonism (*n* = 26)	*p*
Age at examination (years)	57.5 ± 10.5 (38–78)	62.2 ± 10.7 (37–83)	69.7 ± 8.0 (52–84)	< 0.001*
Sex, M/F	14/16	17/22	15/11	0.523
Disease duration (years)	—	8.1 ± 9.4 (0–40)	5.2 ± 4.8 (1–21)	0.867
SARA total score	—	14.1 ± 5.8 (3–26)	—	—
SARA upper limb sub‐score	—	3.0 ± 1.8 (0.5–8)	—	—
ICARS total score	—	36.3 ± 14.9 (15–72)	—	—
ICARS upper limb sub‐score	—	9.7 ± 4.9 (2–25)	—	—

*Note:* Data represent mean ± standard deviation (range). * indicates statistical significance at *p* < 0.05.

Abbreviations: —, not applicable; ICARS, International Cooperative Ataxia Rating Scale; SARA, Scale for the Assessment and Rating of Ataxia.

Additional subgroup‐specific demographic and clinical characteristics are presented (Tables S1 and S2) for patients with MSA‐C and SCA6 and for those with PD, PSP, and MSA‐P, respectively.

### 3.2 Virtual Reality‐Based Measurement Parameters

**FIGURE 3:**
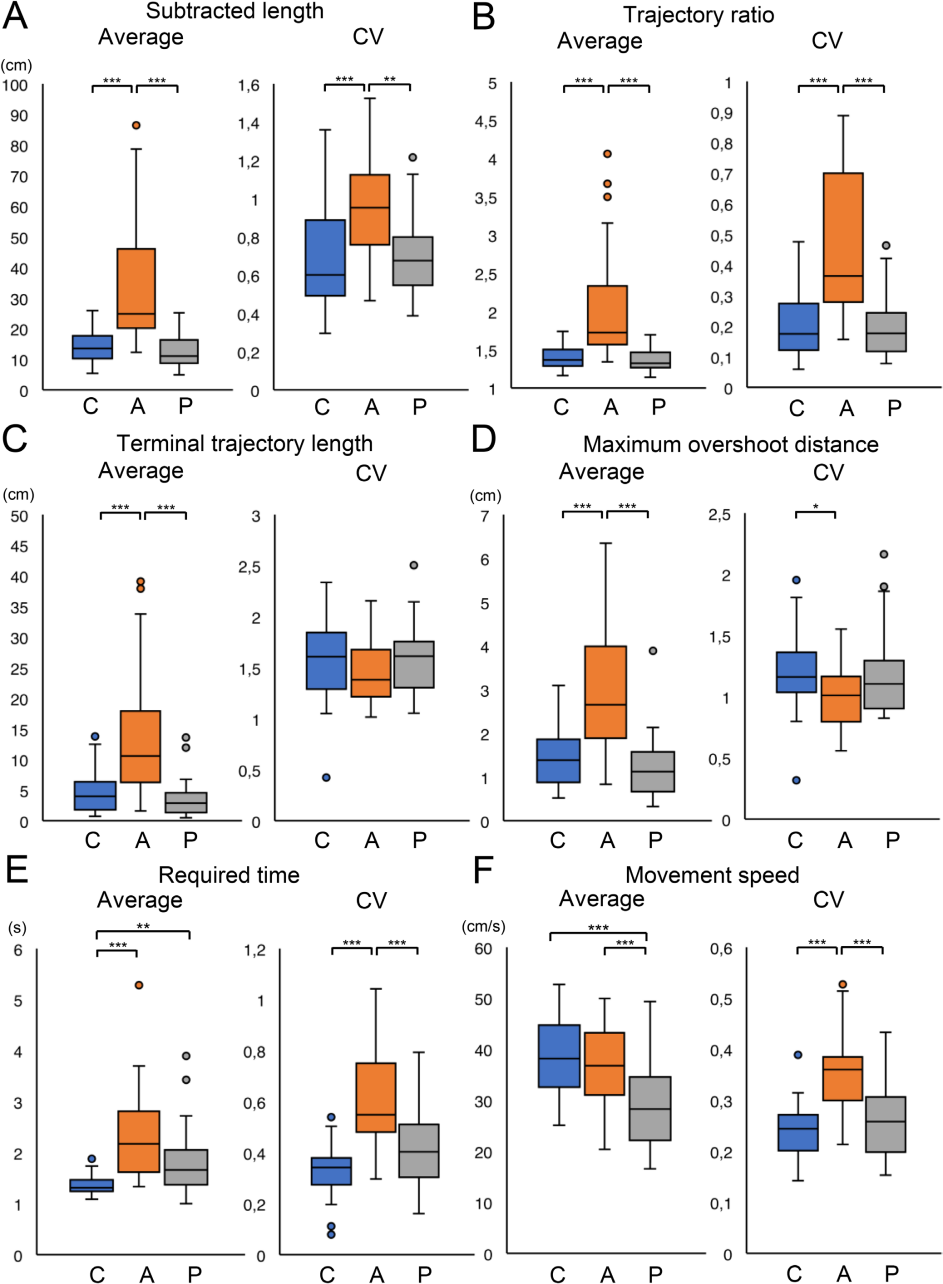
Boxplots of the values of each parameter and the respective CVs measured using the virtual reality device for participants in the control, ataxia, and Parkinsonism groups. Boxplots for the subtracted length and its CV (A), trajectory ratio and its CV (B), terminal trajectory length and its CV (C), maximum overshoot distance and its CV (D), required time and its CV (E), and movement speed and its CV (F) are shown. C, A, and P below the horizontal axis represented participants in the control, ataxia, and Parkinsonism groups. Asterisks indicate significant differences (*p < 0.05, **p < 0.01, ***p < 0.001). CV, coefficient of variation.

The results and the CVs for each parameter measured using the VR device across the three participant groups are shown (Figure 3). The values of the subtracted length, trajectory ratio, terminal trajectory length, and maximum overshoot distance, and CVs of the subtracted length, trajectory ratio, required time, and movement speed in participants with ataxia were significantly greater than those in the other groups. Participants with ataxia and Parkinsonism required a longer time to complete the evaluation task than control participants did. The value of movement speed in participants with Parkinsonism was significantly slower than that of the controls or patients with ataxia.

### 3.3 Diagnostic Accuracy

**FIGURE 4:**
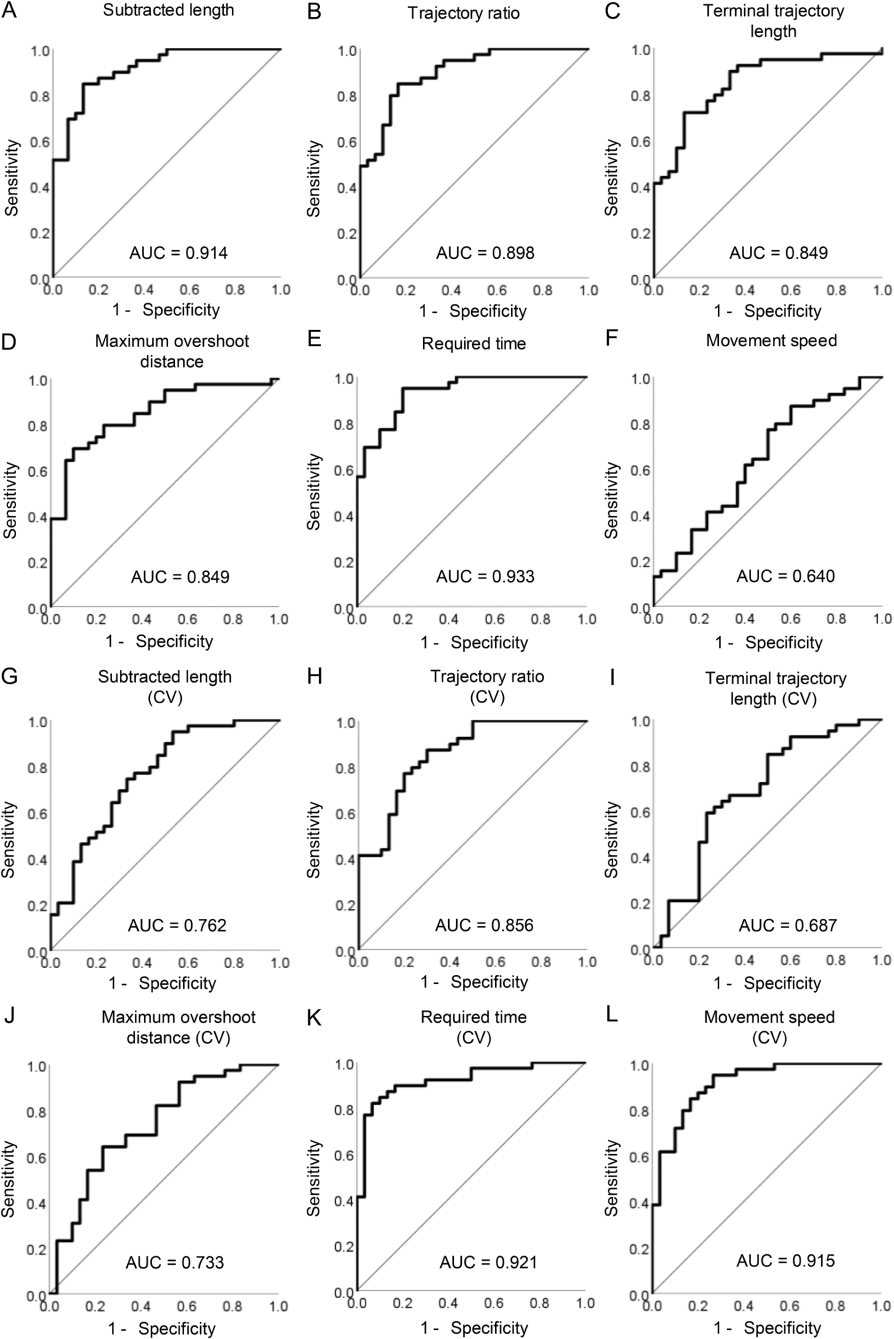
Receiver operating characteristic (ROC) curve analyses for each parameter measured using the virtual reality device to distinguish participants with ataxia from the controls. ROC curves for the values and coefficients of variation of the subtracted length (A, G), trajectory ratio (B, H), terminal trajectory length (C, I), maximum overshoot distance (D, J), required time (E, K), and movement speed (F, L) are shown. The area under the curve is shown for each ROC curve.

The diagnostic accuracy of the VR‐based measurement parameters in distinguishing between participants with ataxia and the controls was assessed using ROC curve analysis (Figure 4). The values of the subtracted length, trajectory ratio, terminal trajectory length, maximum overshoot distance and required time, and the CVs of trajectory ratio, required time, and movement speed demonstrated high accuracy (area under the curve ≥ 0.80) in distinguishing participants with ataxia from the controls.

### 3.4 Correlation Analyses Between Virtual Reality‐Based Parameters and Clinical Scales of Ataxia and Parkinsonism

Correlation analyses were conducted between the VR‐based measurement parameters and the established clinical scales of cerebellar ataxia, including the SARA and ICARS total scores and upper limb sub‐scores (Table 2). All values of the parameters except for movement speed exhibited significant positive correlations with all clinical scales of cerebellar ataxia (Figures [Link], [Link], [Link], [Link]). The CVs of trajectory ratio, required time, movement speed, and the subtracted length showed significant positive correlations with all clinical scales of cerebellar ataxia, except for the SARA total score. Correlation analyses between the parameters measured using the VR device and the total UPDRS scores in participants with PD are shown (Table S3). While one parameter (the CV of the maximum overshoot distance) showed a slightly statistically significant correlation with the UPDRS scores, no consistent or clinically meaningful associations were observed overall.

**TABLE 2:** Correlation analyses between parameters measured using the VR device and SARA and ICARS total scores and upper limb sub‐scores in participants with ataxia.

	SARA total score		SARA upper limb sub‐score		ICARS total score		ICARS upper limb sub‐score	
	*r*	*p*	*r*	*p*	*r*	*p*	*r*	*p*
Subtracted length	0.569	< 0.001*	0.719	< 0.001*	0.696	< 0.001*	0.602	< 0.001*
Trajectory ratio	0.608	< 0.001*	0.711	< 0.001*	0.701	< 0.001*	0.594	< 0.001*
Terminal trajectory length	0.531	< 0.001*	0.657	< 0.001*	0.597	< 0.001*	0.470	0.003*
Maximum overshoot distance	0.497	0.001*	0.609	< 0.001*	0.563	< 0.001*	0.435	0.006*
Required time	0.691	< 0.001*	0.801	< 0.001*	0.706	< 0.001*	0.646	< 0.001*
Movement speed	−0.252	0.122	−0.176	0.284	−0.140	0.395	−0.124	0.450
Subtracted length (CV)	0.305	0.059	0.409	0.010*	0.401	0.011*	0.335	0.037*
Trajectory ratio (CV)	0.532	< 0.001*	0.673	< 0.001*	0.647	< 0.001*	0.543	< 0.001*
Terminal trajectory length (CV)	0.001	0.996	−0.135	0.413	−0.074	0.654	−0.048	0.771
Maximum overshoot distance (CV)	−0.140	0.396	−0.324	0.044*	−0.240	0.141	−0.216	0.186
Required time (CV)	0.481	0.002*	0.658	< 0.001*	0.537	< 0.001*	0.438	0.005*
Movement speed (CV)	0.374	0.019*	0.488	0.002*	0.414	0.009*	0.327	0.042*

* indicates statistical significance at *p* < 0.05.

Abbreviations: CV, coefficient of variation; ICARS, International Cooperative Ataxia Rating Scale; SARA, Scale for the Assessment and Rating of Ataxia; VR, virtual reality.

### 3.5 Score Changes of Virtual Reality‐Based Measurement Parameters in Three Serial Trials

**FIGURE 5:**
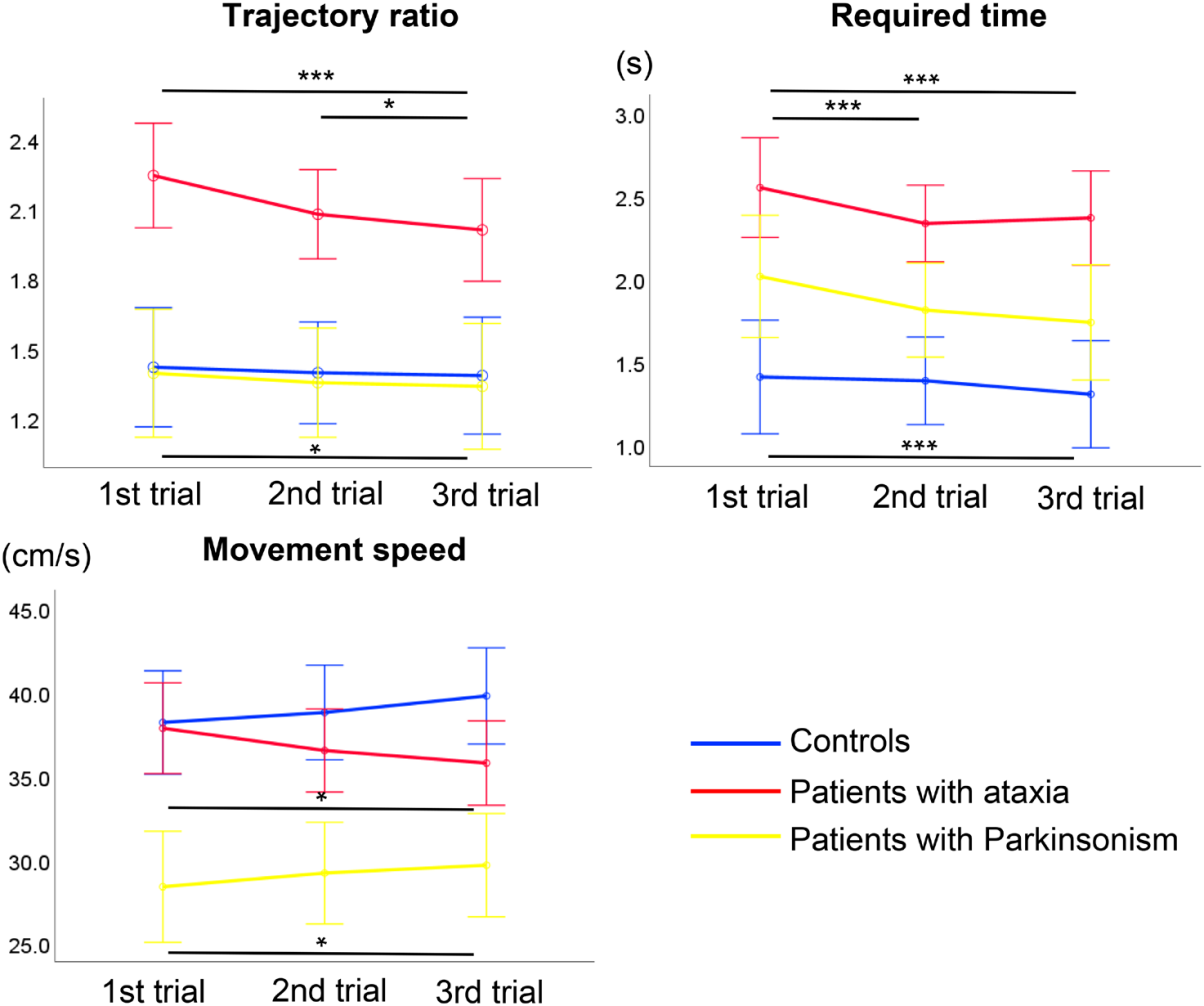
Score changes of three parameters measured using the VR device in three serial trials in participants in the control, ataxia, and Parkinsonism groups. Blue, red, and yellow lines represent the results of the participants in the control, ataxia, and Parkinsonism groups, respectively. Asterisks indicate significant differences (*p < 0.05, ***p < 0.001).

The changes in scores of three VR‐based measurement parameters (trajectory ratio, required time, and movement speed) in the first, second, and third trials for participants in the control, ataxia, and Parkinsonism groups are shown (Figure 5). Participants with ataxia showed a significant reduction in the trajectory ratio from the first to the third trial, whereas no notable change was observed in the controls or those with Parkinsonism. The required time decreased significantly across trials among the controls and participants with Parkinsonism, but no similar trend was observed in those with ataxia. Movement speed increased across trials among the controls and participants with Parkinsonism but decreased in those with ataxia, exhibiting an opposite trend compared with that in the non‐ataxia groups. The statistical results of multiple comparisons between the first, second, and third trials in the three groups are shown (Table S4).

In the sub‐analysis, participants with MSA‐C and those with SCA6 exhibited trends similar to the total participants with ataxia, with participants with MSA‐C showing a significant decrease in trajectory ratio and required time across trials (Figures S6 and S7). The statistical results of multiple comparisons between the first, second, and third trials in participants with MSA‐C and those with SCA6 are shown (Table S5).

## 4 Discussion

In this study, we developed a novel VR‐based system for the quantitative and objective evaluation of upper limb ataxia. Almost all parameters measured using the evaluation program “virtual nose‐finger test” significantly correlated with the upper limb sub‐score of the SARA and the ICARS, and the total score of both clinical scales. In addition, VR‐based parameters can effectively distinguish participants with ataxia from the control participants; therefore, these parameters could serve as reliable and useful clinical biomarkers for evaluating upper limb ataxia. Notably, three parameters, including trajectory ratio, required time (defined as the average time required for each one‐way movement of the participant's index fingertip), and the CV of movement speed (defined as the average speed of upper limb movement) were suggested as sensitive indicators for evaluating upper limb ataxia in patients with cerebellar ataxia.

Cerebellar ataxia is characterized by spatial and temporal movement errors, which have been quantified using various methods [32, 33]. In our study, we assessed six VR‐based parameters, including four spatial (subtracted length, trajectory ratio, terminal trajectory length, and maximum overshoot distance) and two temporal (required time and movement speed) parameters. In the spatial evaluation, participants with ataxia exhibited significantly higher values of subtracted length and trajectory ratio than the controls, and those with Parkinsonism did. These results suggested that participants with ataxia were forced to have excessive trajectories and inefficient movement. Moreover, the CVs for these parameters were greater in participants with ataxia than in participants in the other groups, suggesting that participants with ataxia exhibited greater fluctuations in upper limb movements than those in the other groups did. These findings were consistent with the previous studies evaluating the pointing task in upper limb movements, showing that the spatial trajectories of fingertip movements in patients with cerebellar ataxia were more circuitous and irregular than those in healthy controls [34, 35]. Another study using the optoelectronic system with passive markers suggested that finger movements in patients with cerebellar ataxia were more fragmented and exhibited more frequent directional changes than did those in healthy controls [36]. Furthermore, in a study in which upper limb reaching movements were assessed, the findings showed that upper limb movements of patients with cerebellar ataxia were more variable than those of healthy controls [22]. The subtracted length and trajectory ratio were calculated based on the shortest length, defined as the straight‐line distance between the base point and the target point. A previous study in which a pen‐like sensor device was used revealed that deviations from ideal upper limb trajectories provided a more sensitive measure for assessing the severity of upper limb ataxia [37]. Our results were consistent with these studies and suggested that both spatial parameters might reflect decomposition and dysmetria, which are characteristics of patients with cerebellar ataxia.

Two other spatial parameters, the terminal trajectory length and maximum overshoot distance, were also greater in participants with ataxia than in the other groups. These two parameters were measured during the final phase when the target point was reached. Therefore, these findings suggested that participants with ataxia had difficulty reaching the target, reflecting impaired endpoint accuracy and hypermetria in upper limb movements. A previous study reported that the spatial variability of upper limb movements in patients with cerebellar ataxia was particularly pronounced during the final phase of movement [34]. This finding suggested that individuals with cerebellar ataxia may compensate for spatial inaccuracy through excessive adjustments. Conversely, some previous studies have reported no significant differences in the endpoint accuracy between patients with cerebellar ataxia and healthy controls [36, 38]. These conflicting results may be attributed to variations in evaluation methods. Our VR‐based assessment system was used to measure endpoint accuracy in three dimensions, enabling the evaluation of hypermetria in the anterior–posterior direction, whereas previous studies assessed endpoint accuracy in only two dimensions.

In addition, our results suggested that temporal parameters, including required time and movement speed, were reliable and sensitive clinical biomarkers for evaluating upper limb ataxia. Participants with ataxia and those with Parkinsonism took a longer time to perform the evaluation task than the controls did; however, participants with ataxia exhibited a greater CV of the required time. Furthermore, movement speed was significantly higher in participants with ataxia than in those with Parkinsonism, and the CV of movement speed was more pronounced in participants with ataxia than in the other groups. Considering these results, those with ataxia exhibited increased variability in upper limb speed, contributing to prolonged execution time and increased temporal variability. Previous studies revealed that the execution times of various tasks were longer in patients with cerebellar ataxia than in healthy controls [22, 35, 36, 39]. In addition, previous studies in which circle drawing and letter writing tasks were assessed have revealed increased temporal variability in patients with cerebellar ataxia [40, 41]. A touchscreen‐based assessment of the nose‐finger test similarly reported that participants with ataxia exhibited more arrhythmic and variable upper limb movement speeds than the controls [38]. Furthermore, the CV of upper limb speed was indicated as a more useful and sensitive marker to assess cerebellar ataxia severity than average movement speed [42]. These findings were also consistent with our results.

To further examine whether VR‐based parameters could be useful for assessing motor adaptation in the cerebellum, we compared three measurement parameters (trajectory ratio, required time, and movement speed) in three successive trials. Our results indicated that participants with ataxia exhibited distinct patterns of motor adaptation compared with those of the control and Parkinsonism groups. Participants in the control and Parkinsonism groups demonstrated a reduction in their required time and increased movement speed while maintaining consistent spatial accuracy across trials. In contrast, although participants with ataxia showed improved spatial accuracy across trials, their required time fluctuated, and their movement speed gradually decreased. Similar trends were observed in a sub‐analysis of MSA‐C and SCA6, suggesting a shared compensatory strategy among patients with cerebellar ataxia. Consistent with previous findings, these results indicate that participants with ataxia adopt different strategies by reducing their upper limb speed as a compensatory mechanism to improve spatial accuracy.

The cerebellum is crucial for motor adaptation, and the severity of cerebellar ataxia correlates with impaired motor adaptation in patients with cerebellar ataxia [43, 44]. A study using the treadmill‐based gait analysis reported that patients with cerebellar ataxia failed to adapt their movements as found in healthy controls, with adaptation patterns varying across trials [45]. Additionally, another study reported that patients with cerebellar ataxia compensated for their impairment by reducing movement speed to improve spatial accuracy [46]. Furthermore, spatial adaptation was reported to be easier to achieve than temporal adaptation in patients with cerebellar ataxia [47]. Our results were considered consistent with these previous studies, suggesting that participants with ataxia could adjust for spatial errors across trials but have difficulty with temporal adaptation.

The HMD‐VR‐based evaluation system offers several advantages beyond its capacity to provide highly accurate measurements. Unlike conventional sensor‐based systems that require external cameras, markers, or a fixed recording setup, our VR‐based system is fully portable and markerless, making it feasible for use in various clinical settings, including outpatient and bedside assessments. For example, a previous study conducted using a Kinect‐based system successfully assessed upper limb ataxia with a nose‐finger test and revealed average movement velocity as a sensitive parameter [48]. However, such systems require external depth sensors and relatively large setups, limiting their practical utility in routine outpatient or bedside settings. Furthermore, the immersive and distraction‐free VR environment allows a standardized task with randomized spatial targets. The markerless system also reduces the burden on participants' arms, allowing them to concentrate fully on the evaluation task. A comparative study between VR‐based and conventional training in patients with traumatic brain injury showed the superiority of VR‐based training in enhancing attention [49]. Another benefit of our VR‐based approach was motivation maintenance by providing a strong sense of immersion. For instance, immersive VR‐based training was more effective than non‐immersive training in improving balance function [50].

In addition, this evaluation system can be safely applied to patients with cerebellar ataxia. Notably, adverse effects, such as loss of direction or nausea, have been associated with immersive VR‐based training; however, none of this study's participants experienced simulator sickness [51]. This may be attributed to the simple tasks and short time required for the evaluation. A previous study also reported no significant difference in the incidence of simulator sickness between the HMD‐VR group and the conventional training group during visuomotor adaptation tasks [52]. In summary, our VR‐based evaluation system is considered safe and imposes a low burden on the study participants.

Apart from its clinical utility, our VR‐based system may also serve as a standardized training platform for clinical scales such as the SARA or ICARS. A video‐based SARA training and certification program was successfully developed in a previous study to reduce inter‐rater variability [53]. By offering a reproducible, quantifiable, and immersive simulation of the nose‐finger test, our system has the potential to support clinical education and enhance inter‐rater reliability in clinical scoring, although its educational utility requires further validation studies.

There were some limitations in this study. First, we did not stratify participants with cerebellar ataxia based on disease severity, owing to the relatively small sample size, particularly in patients with advanced stages. Given the heterogeneous progression of cerebellar ataxias, future studies with larger cohorts should be conducted to explore whether the sensitivity of VR‐based parameters differs across various stages of disease severity. Second, although our VR‐based parameters demonstrated strong correlations with established clinical scales and significant group‐level differences, we did not evaluate test–retest reliability or estimate the minimal detectable change. Additionally, it remains unclear whether these parameters are sufficiently sensitive to detect subtle changes in disease severity or to reduce the required sample size in clinical trials compared with using conventional clinical scales, such as the SARA and ICARS. Future studies are needed to validate the psychometric standards of these parameters and to assess their potential utility as endpoints in clinical trials. Third, although each VR‐based parameter was analyzed independently in this study, future research should be conducted to explore the development of a composite index using deep learning. Such an index could serve as a more comprehensive and sensitive assessment of upper limb ataxia and may enhance the evaluation of therapeutic efficacy in clinical trials.

In conclusion, we developed a system using a VR device to evaluate the severity of upper limb ataxia. Our results revealed that parameters measured using the evaluation program's “virtual nose‐finger test” could distinguish participants with ataxia from the controls. The VR‐based assessment could provide a noninvasive, quantitative, and convenient method for evaluating upper limb ataxia. Furthermore, our findings suggested that this system could serve as a neurophysiological biomarker for assessing the severity of cerebellar ataxia. This evaluation system can be applied regardless of a patient's ability to walk, making it a widely accessible tool for assessing upper limb ataxia in clinical settings, including clinical trials of potential treatments for cerebellar ataxia.

## Author Contributions

**Masayuki Sato:** study conceptualization, data acquisition, data analysis, interpretation of results, and drafting of the manuscript. **Takayuki Abe:** development of the VR program, data analysis, and technical support. **Sho Aoki and Setsuki Tsukagoshi:** patient recruitment and data acquisition. **Yasushi Yuminaka:** supervision of VR program development and data analysis, and critical review of the manuscript. **Yoshio Ikeda:** study conception and design, overall supervision, interpretation of results, and critical revision of the manuscript. All authors reviewed the manuscript and approved the final version for submission.

## Conflicts of Interest

The authors declare no conflicts of interest.

## Supporting information


**Figure S1:** Boxplot of ages at examination for participants in the control, ataxia, and Parkinsonism groups. Ages at the examination for participants in the control, ataxia, and Parkinsonism groups are expressed using boxplots. Participants with Parkinsonism were significantly older than the controls and those with ataxia. No significant differences were observed between the control and ataxic groups.

**Figure S2:** Correlation analyses between the SARA total score and each parameter measured using the virtual reality device in participants with ataxia. Correlations between the SARA total score and the values or the CVs of subtracted length (A and G), trajectory ratio (B and H), terminal trajectory length (C and I), maximum overshoot distance (D and J), required time (E and K), and movement speed (F and L) were analyzed in the participants with ataxia. Correlation coefficients and *p*‐values are indicated in each graph. CV, coefficient of variation; SARA, Scale for the Assessment and Rating of Ataxia.

**Figure S3:** Correlation analyses between the SARA upper limb sub‐score and each parameter measured using the virtual reality device in participants with ataxia. Correlations between the SARA upper limb sub‐score and the values or the CVs of subtracted length (A and G), trajectory ratio (B and H), terminal trajectory length (C and I), maximum overshoot distance (D and J), required time (E and K), and movement speed (F and L) were analyzed in participants with ataxia. Correlation coefficients and *p*‐values are indicated in each graph. CV, coefficient of variation; SARA, Scale for the Assessment and Rating of Ataxia.

**Figure S4:** Correlation analyses between the ICARS total score and each parameter measured using the virtual reality device in participants with ataxia. Correlations between the ICARS total score and the values or CVs of subtracted length (A and G), trajectory ratio (B and H), terminal trajectory length (C and I), maximum overshoot distance (D and J), required time (E and K), and movement speed (F and L) were analyzed in participants with ataxia. Correlation coefficients and *p*‐values are indicated in each graph. CV, coefficient of variation; ICARS, International Cooperative Ataxia Rating Scale.

**Figure S5:** Correlation analyses between the ICARS upper limb sub‐score and each parameter measured using the virtual reality device in participants with ataxia. Correlations between the ICARS upper limb sub‐score and the values or CVs of subtracted length (A and G), trajectory ratio (B and H), terminal trajectory length (C and I), maximum overshoot distance (D and J), required time (E and K), and movement speed (F and L) were analyzed in participants with ataxia. Correlation coefficients and *p*‐values are indicated in each graph. CV, coefficient of variation; ICARS, International Cooperative Ataxia Rating Scale.

**Figure S6:** Score changes of three parameters measured using the virtual reality device in three serial trials in participants in the control, MSA‐C, and Parkinsonism groups. The blue, green, and yellow lines represent the results of the participants in the control, MSA‐C, and Parkinsonism groups, respectively. Asterisks indicate significant differences (*p < 0.05, **p < 0.01, ***p < 0.001). MSA‐C, multiple system atrophy with predominant cerebellar ataxia.

**Figure S7:** Score changes of three parameters measured using the virtual reality device in three serial trials in participants in the control, SCA6, and Parkinsonism groups. The blue, orange, and yellow lines express the results of participants in the control, SCA6, and Parkinsonism groups, respectively. Asterisks indicate significant differences (*p < 0.05, **p < 0.01, ***p < 0.001). SCA6, spinocerebellar ataxia type 6.

**Table S1:** Clinical characteristics of the participants with MSA‐C and SCA6.
**Table S2:** Clinical characteristics of the participants with PD, PSP and MSA‐P.
**Table S3:** Correlation analyses between parameters measured by the VR device and the total UPDRS scores in participants with PD.
**Table S4:** Multiple comparisons between the first, second, and third trials of three measurement parameters (trajectory ratio, required time, and movement speed) in participants in the control, ataxic, and Parkinsonism groups.
**Table S5:** Multiple comparisons between the first, second, and third trials of the three measurement parameters (trajectory ratio, required time, and movement speed) in participants with MSA‐C and SCA6.

## Data Availability

The data that support the findings of this study are available on request from the corresponding author. The data are not publicly available due to privacy or ethical restrictions.

## References

[1] T. Klockgether , C. Mariotti , and H. L. Paulson , “Spinocerebellar Ataxia,” Nature Reviews Disease Primers 5 (2019): 24. 10.1038/s41572-019-0074-3 30975995

[2] S. M. Brooker , C. R. Edamakanti , S. M. Akasha , S. H. Kuo , and P. Opal , “Spinocerebellar Ataxia Clinical Trials: Opportunities and Challenges,” Annals of Clinical Translational Neurology 8 (2021): 1543–1556. 34019331 10.1002/acn3.51370 PMC8283160

[3] P. Trouillas , T. Takayanagi , M. Hallett , et al., “International Cooperative Ataxia Rating Scale for Pharmacological Assessment of the Cerebellar Syndrome. The Ataxia Neuropharmacology Committee of the World Federation of Neurology,” Journal of the Neurological Sciences 145 (1997): 205–211. 9094050 10.1016/s0022-510x(96)00231-6

[4] T. Schmitz‐Hübsch , S. T. du Montcel , L. Baliko , et al., “Scale for the Assessment and Rating of Ataxia: Development of a New Clinical Scale,” Neurology 66 (2006): 1717–1720. 16769946 10.1212/01.wnl.0000219042.60538.92

[5] A. Weyer , M. Abele , T. Schmitz‐Hubsch , et al., “Reliability and Validity of the Scale for the Assessment and Rating of Ataxia: A Study in 64 Ataxia Patients,” Movement Disorders 22 (2007): 1633–1637. 17516493 10.1002/mds.21544

[6] I. Yabe , M. Matsushima , H. Soma , R. Basri , and H. Sasaki , “Usefulness of the Scale for Assessment and Rating of Ataxia (SARA),” Journal of the Neurological Sciences 266 (2008): 164–166. 17950753 10.1016/j.jns.2007.09.021

[7] J. A. Saute , K. C. Donis , C. Serrano‐Munuera , et al., “Ataxia Rating Scales–Psychometric Profiles, Natural History and Their Application in Clinical Trials,” Cerebellum 11 (2012): 488–504. 21964941 10.1007/s12311-011-0316-8

[8] D. Bourcier , N. Belair , E. A. Pedneault‐Tremblay , et al., “French Translation and Cross‐Cultural Adaptation of the Scale for the Assessment and Rating of Ataxia,” Cerebellum 22 (2023): 1118–1122. 36208403 10.1007/s12311-022-01484-3

[9] G. Arcuria , C. Marcotulli , R. Amuso , et al., “Developing an Objective Evaluating System to Quantify the Degree of Upper Limb Movement Impairment in Patients With Severe Friedreich's Ataxia,” Neurological Sciences 41 (2020): 1577–1587. 31993871 10.1007/s10072-020-04249-0

[10] H. Tran , K. D. Nguyen , P. N. Pathirana , M. K. Horne , L. Power , and D. J. Szmulewicz , “A Comprehensive Scheme for the Objective Upper Body Assessments of Subjects With Cerebellar Ataxia,” Journal of Neuroengineering and Rehabilitation 17 (2020): 162. 33276783 10.1186/s12984-020-00790-3 PMC7718681

[11] A. Traschutz , A. D. Adarmes‐Gomez , M. Anheim , et al., “Responsiveness of the Scale for the Assessment and Rating of Ataxia and Natural History in 884 Recessive and Early Onset Ataxia Patients,” Annals of Neurology 94 (2023): 470–485. 37243847 10.1002/ana.26712

[12] H. Jacobi , S. T. du Montcel , P. Bauer , et al., “Long‐Term Disease Progression in Spinocerebellar Ataxia Types 1, 2, 3, and 6: A Longitudinal Cohort Study,” Lancet Neurology 14 (2015): 1101–1108. 26377379 10.1016/S1474-4422(15)00202-1

[13] A. Diallo , H. Jacobi , S. Tezenas du Montcel , and T. Klockgether , “Natural History of Most Common Spinocerebellar Ataxia: A Systematic Review and Meta‐Analysis,” Journal of Neurology 268 (2021): 2749–2756. 32266540 10.1007/s00415-020-09815-2

[14] L. Ruano , C. Melo , M. C. Silva , and P. Coutinho , “The Global Epidemiology of Hereditary Ataxia and Spastic Paraplegia: A Systematic Review of Prevalence Studies,” Neuroepidemiology 42 (2014): 174–183. 24603320 10.1159/000358801

[15] F. De Mattei , F. Ferrandes , S. Gallone , et al., “Epidemiology of Spinocerebellar Ataxias in Europe,” Cerebellum 23 (2024): 1176–1183. 37698771 10.1007/s12311-023-01600-x PMC11102384

[16] M. Ferrarin , M. Gironi , L. Mendozzi , R. Nemni , P. Mazzoleni , and M. Rabuffetti , “Procedure for the Quantitative Evaluation of Motor Disturbances in Cerebellar Ataxic Patients,” Medical & Biological Engineering & Computing 43 (2005): 349–356. 16035223 10.1007/BF02345812

[17] M. Germanotta , G. Vasco , M. Petrarca , et al., “Robotic and Clinical Evaluation of Upper Limb Motor Performance in Patients With Friedreich's Ataxia: An Observational Study,” Journal of Neuroengineering and Rehabilitation 12 (2015): 41. 25900021 10.1186/s12984-015-0032-6 PMC4448881

[18] S. Tsukagoshi , M. Furuta , K. Hirayanagi , et al., “Noninvasive and Quantitative Evaluation of Movement Disorder Disability Using an Infrared Depth Sensor,” Journal of Clinical Neuroscience 71 (2020): 135–140. 31501004 10.1016/j.jocn.2019.08.101

[19] E. Peri , D. Panzeri , E. Beretta , G. Reni , S. Strazzer , and E. Biffi , “Motor Improvement in Adolescents Affected by Ataxia Secondary to Acquired Brain Injury: A Pilot Study,” BioMed Research International 2019 (2019): 8967138. 31886263 10.1155/2019/8967138 PMC6899307

[20] E. Lo Voi , G. C. Basile , A. Bramanti , et al., “Cerebellar Atrophy Associated With Primary Sjögren's Syndrome: Diagnosis, Therapy, and Virtual Reality Rehabilitation: A Case Report,” Innovations in Clinical Neuroscience 18 (2021): 11–17. PMC8667698 34980988

[21] K. Takimoto , K. Omon , Y. Murakawa , and H. Ishikawa , “Case of Cerebellar Ataxia Successfully Treated by Virtual Reality‐Guided Rehabilitation,” BML Case Reports 14 (2021): e242287. 10.1136/bcr-2021-242287 PMC8112436 33972306

[22] A. S. Therrien , M. A. Statton , and A. J. Bastian , “Reinforcement Signaling Can Be Used to Reduce Elements of Cerebellar Reaching Ataxia,” Cerebellum 20 (2021): 62–73. 32880848 10.1007/s12311-020-01183-x PMC7927977

[23] H. Chang , S. H. Woo , S. Kang , et al., “A Curtailed Task for Quantitative Evaluation of Visuomotor Adaptation in the Head‐Mounted Display Virtual Reality Environment,” Frontiers in Psychiatry 13 (2022): 963303. 36895426 10.3389/fpsyt.2022.963303 PMC9989973

[24] R. B. Postuma , D. Berg , M. Stern , et al., “MDS Clinical Diagnostic Criteria for Parkinson's Disease,” Movement Disorders 30 (2015): 1591–1601. 26474316 10.1002/mds.26424

[25] G. U. Hoglinger , G. Respondek , M. Stamelou , et al., “Clinical Diagnosis of Progressive Supranuclear Palsy: The Movement Disorder Society Criteria,” Movement Disorders 32 (2017): 853–864. 28467028 10.1002/mds.26987 PMC5516529

[26] G. K. Wenning , I. Stankovic , L. Vignatelli , et al., “The Movement Disorder Society Criteria for the Diagnosis of Multiple System Atrophy,” Movement Disorders 37 (2022): 1131–1148. 35445419 10.1002/mds.29005 PMC9321158

[27] A. Carnevale , I. Mannocchi , M. S. H. Sassi , et al., “Virtual Reality for Shoulder Rehabilitation: Accuracy Evaluation of Oculus Quest 2,” Sensors (Basel) 22 (2022): 5511. 35898015 10.3390/s22155511 PMC9332705

[28] C. M. Craig , J. Stafford , A. Egorova , C. McCabe , and M. Matthews , “Can We Use the Oculus Quest VR Headset and Controllers to Reliably Assess Balance Stability?,” Diagnostics (Basel) 12 (2022): 1409. 35741219 10.3390/diagnostics12061409 PMC9221913

[29] S. R. Fahn , C. D. Marsden , D. B. Calne , and M. Goldstein , “Unified Parkinson's Disease Rating Scale,” in Recent Developments in Parkinson's Disease, vol. 2, ed. S. Fahn , C. D. Marsden , D. B. Calne , and M. Goldstein (Macmillan, 1987), 153–163.

[30] G. K. Wenning , F. Tison , K. Seppi , et al., “Development and Validation of the Unified Multiple System Atrophy Rating Scale (UMSARS),” Movement Disorders 19 (2004): 1391–1402. 15452868 10.1002/mds.20255

[31] L. I. Golbe and P. A. Ohman‐Strickland , “A Clinical Rating Scale for Progressive Supranuclear Palsy,” Brain 130 (2007): 1552–1565. 17405767 10.1093/brain/awm032

[32] M. Manto , “Mechanisms of Human Cerebellar Dysmetria: Experimental Evidence and Current Conceptual Bases,” Journal of Neuroengineering and Rehabilitation 6 (2009): 10. 19364396 10.1186/1743-0003-6-10 PMC2679756

[33] A. J. Bastian , “Learning to Predict the Future: The Cerebellum Adapts Feedforward Movement Control,” Current Opinion in Neurobiology 16 (2006): 645–649. 17071073 10.1016/j.conb.2006.08.016

[34] B. L. Day , P. D. Thompson , A. E. Harding , and C. D. Marsden , “Influence of Vision on Upper Limb Reaching Movements in Patients With Cerebellar Ataxia,” Brain 121 (1998): 357–372. 9549511 10.1093/brain/121.2.357

[35] F. Marini , C. Chwastek , M. Romei , et al., “Quantitative Evaluation Protocol for Upper Limb Motor Coordination Analysis in Patients With Ataxia,” Annual International Conference of the IEEE Engineering in Medicine and Biology Society 2010 (2010): 6633–6636. 21096730 10.1109/IEMBS.2010.5627144

[36] F. Menegoni , E. Milano , C. Trotti , et al., “Quantitative Evaluation of Functional Limitation of Upper Limb Movements in Subjects Affected by Ataxia,” European Journal of Neurology 16 (2009): 232–239. 19146643 10.1111/j.1468-1331.2008.02396.x

[37] Y. Kishimoto , A. Hashizume , Y. Imai , et al., “Quantitative Evaluation of Upper Limb Ataxia in Spinocerebellar Ataxias,” Annals of Clinical Translational Neurology 9 (2022): 529–539. 35293156 10.1002/acn3.51528 PMC8994984

[38] J. Lipponen , A. Tiulpin , K. Majamaa , and H. Rusanen , “Quantification of Upper Limb Movements in Patients With Hereditary or Idiopathic Ataxia,” Cerebellum 22 (2023): 1182–1191. 36269527 10.1007/s12311-022-01485-2 PMC10657283

[39] G. Arcuria , C. Marcotulli , C. Galasso , F. Pierelli , and C. Casali , “15‐White Dots APP‐Coo‐Test: A Reliable Touch‐Screen Application for Assessing Upper Limb Movement Impairment in Patients With Cerebellar Ataxias,” Journal of Neurology 266 (2019): 1611–1622. 30955123 10.1007/s00415-019-09299-9

[40] J. E. Schlerf , R. M. Spencer , H. N. Zelaznik , and R. B. Ivry , “Timing of Rhythmic Movements in Patients With Cerebellar Degeneration,” Cerebellum 6 (2007): 221–231. 17786818 10.1080/14734220701370643

[41] Y. Fujisawa and Y. Okajima , “Characteristics of Handwriting of People With Cerebellar Ataxia: Three‐Dimensional Movement Analysis of the Pen Tip, Finger, and Wrist,” Physical Therapy 95 (2015): 1547–1558. 25953596 10.2522/ptj.20140118

[42] D. Hermle , R. Schubert , P. Barallon , et al., “Multifeature Quantitative Motor Assessment of Upper Limb Ataxia Including Drawing and Reaching,” Annals of Clinical Translational Neurology 11 (2024): 1097–1109. 38590028 10.1002/acn3.52024 PMC11093241

[43] M. Ito , “Cerebellar Circuitry as a Neuronal Machine,” Progress in Neurobiology 78 (2006): 272–303. 16759785 10.1016/j.pneurobio.2006.02.006

[44] M. A. Statton , A. Vazquez , S. M. Morton , E. V. L. Vasudevan , and A. J. Bastian , “Making Sense of Cerebellar Contributions to Perceptual and Motor Adaptation,” Cerebellum 17 (2018): 111–121. 28840476 10.1007/s12311-017-0879-0 PMC5826770

[45] M. K. Rand , D. A. Wunderlich , P. E. Martin , G. E. Stelmach , and J. R. Bloedel , “Adaptive Changes in Responses to Repeated Locomotor Perturbations in Cerebellar Patients,” Experimental Brain Research 122 (1998): 31–43. 9772109 10.1007/s002210050488

[46] M. Manto , J. M. Bower , A. B. Conforto , et al., “Consensus Paper: Roles of the Cerebellum in Motor Control—The Diversity of Ideas on Cerebellar Involvement in Movement,” Cerebellum 11 (2012): 457–487. 22161499 10.1007/s12311-011-0331-9 PMC4347949

[47] L. A. Malone and A. J. Bastian , “Thinking About Walking: Effects of Conscious Correction Versus Distraction on Locomotor Adaptation,” Journal of Neurophysiology 103 (2010): 1954–1962. 20147417 10.1152/jn.00832.2009 PMC2853281

[48] T. Honda , H. Mitoma , H. Yoshida , et al., “Assessment and Rating of Motor Cerebellar Ataxias With the Kinect v2 Depth Sensor: Extending Our Appraisal,” Frontiers in Neurology 11 (2020): 179. 32218767 10.3389/fneur.2020.00179 PMC7078683

[49] J. H. Shin and E. Jeong , “Virtual Reality‐Based Music Attention Training for Acquired Brain Injury: A Protocol for Randomized Cross‐Over Trial,” Frontiers in Neurology 14 (2023): 1192181. 37638184 10.3389/fneur.2023.1192181 PMC10450247

[50] M. Liu , K. Zhou , Y. Chen , L. Zhou , D. Bao , and J. Zhou , “Is Virtual Reality Training More Effective Than Traditional Physical Training on Balance and Functional Mobility in Healthy Older Adults? A Systematic Review and Meta‐Analysis,” Frontiers in Human Neuroscience 16 (2022): 843481. 35399351 10.3389/fnhum.2022.843481 PMC8984187

[51] L. Simón‐Vicente , S. Rodríguez‐Cano , V. Delgado‐Benito , et al., “A Systematic Literature Review of Adverse Effects Related to Virtual Reality,” Neurología 39 (2024): 701–709. 39396266 10.1016/j.nrleng.2022.04.007

[52] J. M. Anglin , T. Sugiyama , and S. L. Liew , “Visuomotor Adaptation in Head‐Mounted Virtual Reality Versus Conventional Training,” Scientific Reports 7 (2017): 45469. 28374808 10.1038/srep45469 PMC5379618

[53] M. Grobe‐Einsler , A. T. Amin , J. Faber , H. Völkel , M. Synofzik , and T. Klockgether , “Scale for the Assessment and Rating of Ataxia (SARA): Development of a Training Tool and Certification Program,” Cerebellum 23 (2024): 877–880. 36922437 10.1007/s12311-023-01543-3 PMC11102411

