# Supplementary figures and images for: Quantitative Assessment of Upper Limb Ataxia Using a Virtual Reality‐Based Evaluation System
Source: Ann Clin Transl Neurol. 2025 Oct 2;13(1):180–92. doi: 10.1002/acn3.70215 (PMC12790176; doi:10.1002/acn3.70215)

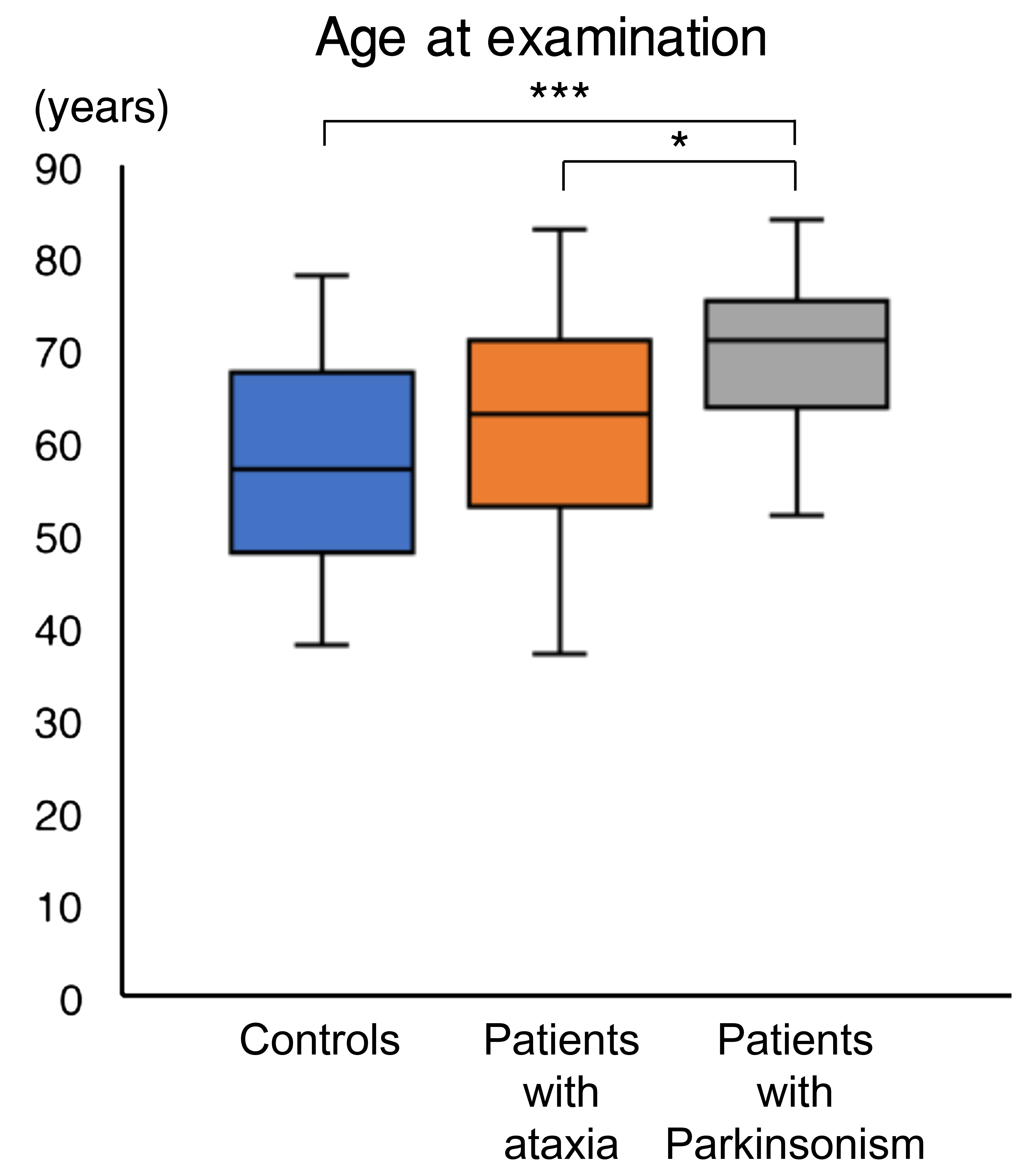

Supplement: Supplementary file 1 — Figure S1: Boxplot of ages at examination for participants in the control, ataxia, and Parkinsonism groups. Ages at the examination for participants in the control, ataxia, and Parkinsonism groups are expressed using boxplots. Participants with Parkinsonism were significantly older than the controls and those with ataxia. No significant differences were observed between the control and ataxic groups. [file ACN3-13-180-s008.tif]

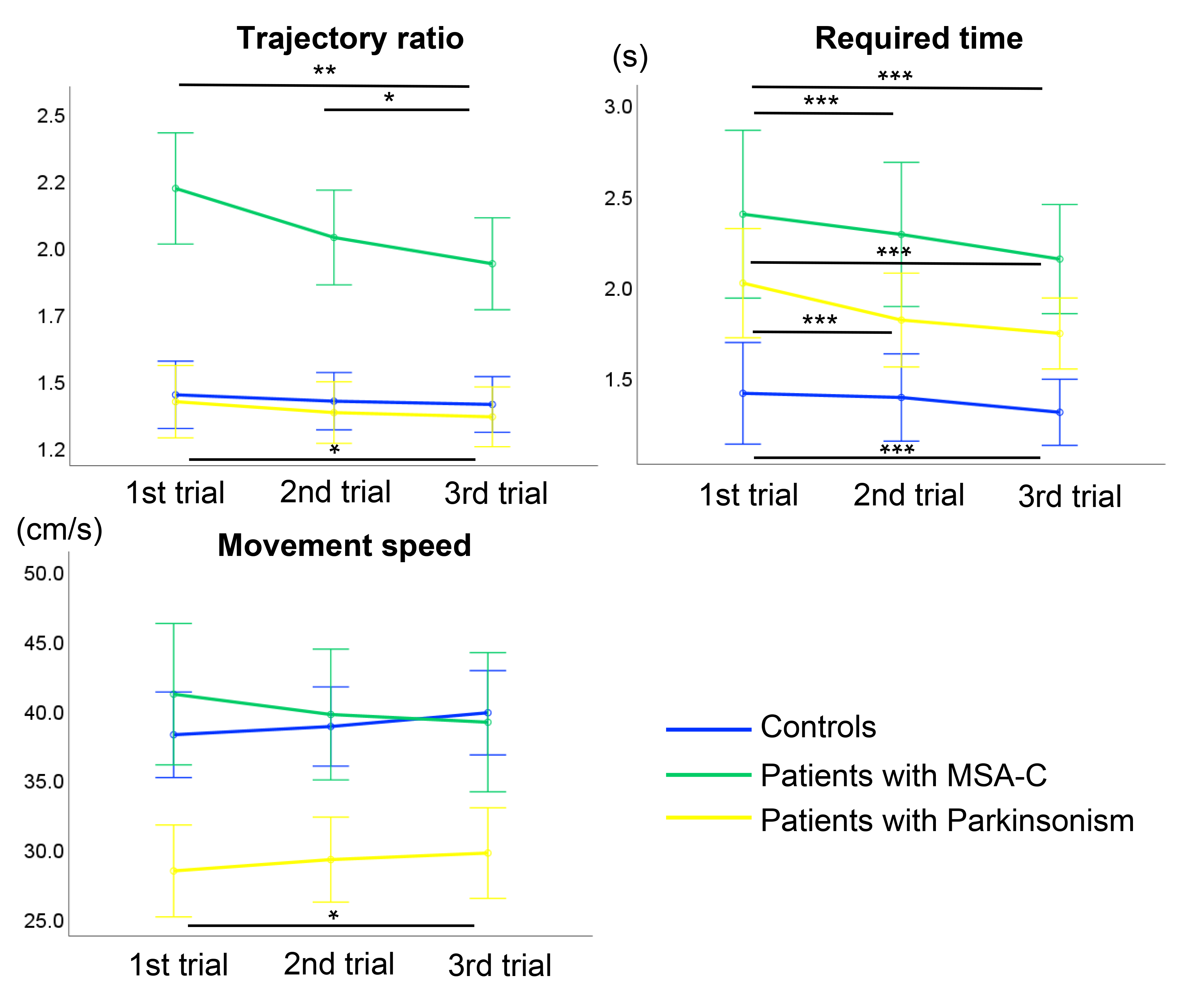

Supplement: Supplementary file 6 — Figure S6: Score changes of three parameters measured using the virtual reality device in three serial trials in participants in the control, MSA‐C, and Parkinsonism groups. The blue, green, and yellow lines represent the results of the participants in the control, MSA‐C, and Parkinsonism groups, respectively. Asterisks indicate significant differences (*p < 0.05, **p < 0.01, ***p < 0.001). MSA‐C, multiple system atrophy with predominant cerebellar ataxia. [file ACN3-13-180-s006.tif]

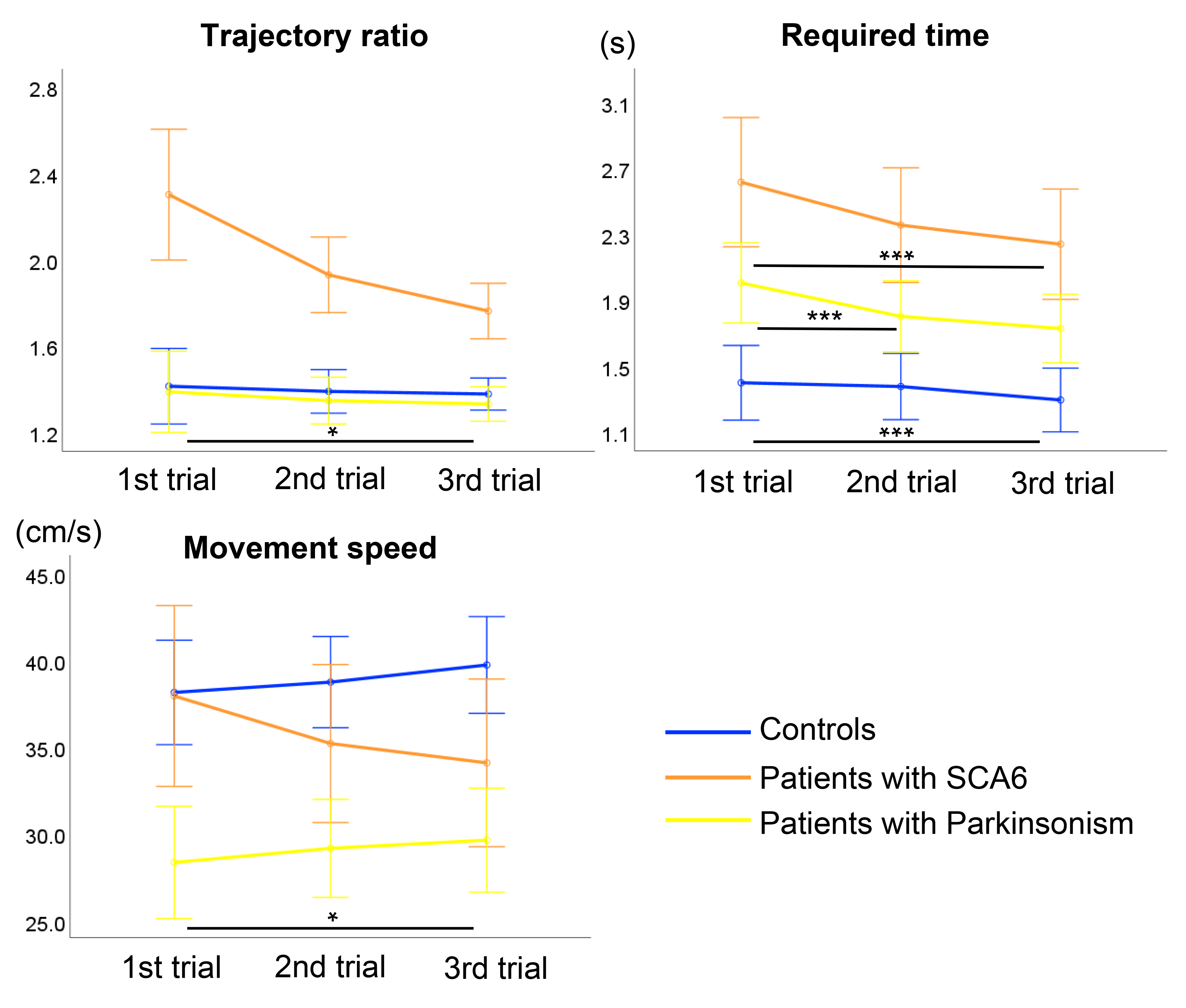

Supplement: Supplementary file 7 — Figure S7: Score changes of three parameters measured using the virtual reality device in three serial trials in participants in the control, SCA6, and Parkinsonism groups. The blue, orange, and yellow lines express the results of participants in the control, SCA6, and Parkinsonism groups, respectively. Asterisks indicate significant differences (*p < 0.05, **p < 0.01, ***p < 0.001). SCA6, spinocerebellar ataxia type 6. [file ACN3-13-180-s002.tif]
