# Supplementary material for: Quantitative Assessment of Upper Limb Ataxia Using a Virtual Reality‐Based Evaluation System
Source: Ann Clin Transl Neurol. 2025 Oct 2;13(1):180–92. doi: 10.1002/acn3.70215 (PMC12790176; doi:10.1002/acn3.70215)
Supplement: Supplementary file 8 — Table S1: Clinical characteristics of the participants with MSA‐C and SCA6. Table S2: Clinical characteristics of the participants with PD, PSP and MSA‐P. Table S3: Correlation analyses between parameters measured by the VR device and the total UPDRS scores in participants with PD. Table S4: Multiple comparisons between the first, second, and third trials of three measurement parameters (trajectory ratio, required time, and movement speed) in participants in the control, ataxic, and Parkinsonism groups. Table S5: Multiple comparisons between the first, second, and third trials of the three measurement parameters (trajectory ratio, required time, and movement speed) in participants with MSA‐C and SCA6. [file ACN3-13-180-s007.docx]

**Supplementary Table 1.**

|  | Patients with MSA-C  (n = 11) | Patients with SCA6  (n = 10) | *p*-value |
| --- | --- | --- | --- |
| Age at examination (years) | 61.7 ± 7.6  (50–74) | 61.7 ± 12.0  (45–83) | 0.969 |
| Sex, M/F | 4/7 | 4/6 | 1.000 |
| Disease duration (years) | 2.7 ± 2.1  (0.6–8.3) | 11.6 ± 9.8  (1.1–30.8) | 0.036* |
| SARA total score | 12.9 ± 6.0  (6.5–25.5) | 13.8 ± 6.7  (3–25) | 0.756 |
| SARA upper limb subscore | 3.0 ± 1.8  (1–7) | 3.0 ± 2.1  (1–8) | 0.918 |
| ICARS total score | 30.6 ± 16.0  (18–72) | 36.1 ± 16.3  (15–70) | 0.349 |
| ICARS upper limb subscore | 10.0 ± 5.5  (3–22) | 8.6 ± 5.6  (3–22) | 0.468 |
| UMSARS Part1 | 12.1 ± 5.6  (6–26) | - | - |
| UMSARS Part2 | 14.2 ± 9.7  (4-33) | - | - |

Clinical characteristics of the participants with MSA-C and SCA6

Abbreviations: MSA-C, multiple system atrophy with predominant cerebellar ataxia; SCA6, spinocerebellar ataxia type 6; SARA, Scale for the Assessment and Rating of Ataxia; ICARS, International Cooperative Ataxia Rating Scale; UMSARS, the Unified Multiple System Atrophy Rating Scale. Data represent mean ± standard deviation (range).

**Supplementary Table 2.**

|  | Patients with PD  (n = 18) | Patients with PSP  (n = 5) | Patients with MSA-P  (n = 3) |
| --- | --- | --- | --- |
| Age at examination (years) | 70.3 ± 8.2  (59–84) | 72.0 ± 4.3  (65–76) | 63.0 ± 10.5  (52–73) |
| Sex, M/F | 10/8 | 4/1 | 1/2 |
| Disease duration (years) | 6.2 ± 5.4  (1.7–21.3) | 3.6 ± 1.4  (2.0–5.6) | 1.7 ± 0.7  (1.0–2.5) |
| UPDRS | 23.7 ± 9.2  (11–43) | - | - |
| PSPRS | - | 26.6 ± 8.2  (19–36) | - |
| UMSARS Part1 | - | - | 16.3 ± 10.0  (6–26) |
| UMSARS Part2 | - | - | 22.0 ± 10.1  (11–31) |

Clinical characteristics of the participants with PD, PSP and MSA-P

Abbreviations: PD, Parkinson’s disease; PSP, progressive supranuclear palsy; MSA-P, multiple system atrophy with predominant parkinsonism; UPDRS, the Unified Parkinson's Disease Rating Scale; PSPRS, the Progressive Supranuclear Palsy Rating Scale; UMSARS, the Unified Multiple System Atrophy Rating Scale. Data represent mean ± standard deviation (range).

**Supplementary Table 3.**

Correlation analyses between parameters measured by the VR device and the total UPDRS scores in participants with PD

|  | **UPDRS total score** | |
| --- | --- | --- |
|  | *r* | *p*-value |
| **Subtracted length** | 0.154 | 0.542 |
| **Trajectory ratio** | 0.208 | 0.408 |
| **Terminal trajectory length** | -0.096 | 0.704 |
| **Maximum overshoot distance** | -0.025 | 0.922 |
| **Required time** | 0.195 | 0.437 |
| **Movement speed** | -0.109 | 0.668 |
| **Subtracted length (CV)** | 0.173 | 0.493 |
| **Trajectory ratio (CV)** | 0.195 | 0.437 |
| **Terminal trajectory length (CV)** | -0.240 | 0.338 |
| **Maximum overshoot distance (CV)** | -0.479 | 0.045* |
| **Required time (CV)** | 0.192 | 0.445 |
| **Movement speed (CV)** | 0.171 | 0.499 |

Abbreviations: VR, virtual reality; UPDRS, the Unified Parkinson's Disease Rating Scale; PD, Parkinson’s disease; CV, coefficient of variation.

**Supplementary Table 4.**

| **Trajectory ratio** | **Group**  **difference** | **1st trial**  **vs. 2nd trial** | **2nd trial**  **vs. 3rd trial** | **1st trial**  **vs. 3rd trial** |
| --- | --- | --- | --- | --- |
|  | *p*- value | *p*- value | *p*- value | *p*- value |
| Controls | 0.067 | NS | NS | NS |
| Patients with Ataxia | < 0.001* | 0.028* | 0.339 | < 0.001* |
| Patients with  Parkinsonism | 0.019* | 0.214 | 0.995 | 0.017* |

Multiple comparisons between the first, second, and third trials of three measurement parameters (trajectory ratio, required time, and movement speed) in participants in the control, ataxic, and parkinsonism groups

| **Required time** | **Group**  **difference** | **1st trial**  **vs. 2nd trial** | **2nd trial**  **vs. 3rd trial** | **1st trial**  **vs. 3rd trial** |
| --- | --- | --- | --- | --- |
|  | *p*- value | *p*- value | *p*- value | *p*- value |
| Controls | < 0.001* | 0.117 | 0.117 | < 0.001* |
| Patients with Ataxia | 0.209 | NS | NS | NS |
| Patients with Parkinsonism | < 0.001* | 0.001* | 1.000 | < 0.001* |

| **Movement speed** | **Group**  **difference** | **1st trial**  **vs. 2nd trial** | **2nd trial**  **vs. 3rd trial** | **1st trial**  **vs. 3rd trial** |
| --- | --- | --- | --- | --- |
|  | *p*- value | *p*-value | *p*- value | *p*- value |
| Controls | 0.088 | NS | NS | NS |
| Patients with Ataxia | 0.018* | 0.339 | 0.639 | 0.014* |
| Patients with Parkinsonism | 0.019* | 0.214 | 0.995 | 0.017* |

Abbreviations: NS, not significant

**Supplementary Table 5.**

| **Trajectory ratio** | **Group**  **difference** | **1st trial**  **vs. 2nd trial** | **2nd trial**  **vs. 3rd trial** | **1st trial**  **vs. 3rd trial** |
| --- | --- | --- | --- | --- |
|  | *p*-value | *p*-value | *p*-value | *p*-value |
| Patients with MSA-C | 0.009* | 0.033* | 0.394 | 0.003* |
| Patients with SCA6 | 0.082 | NS | NS | NS |

Multiple comparisons between the first, second, and third trials of the three measurement parameters (trajectory ratio, required time, and movement speed) in participants with MSA-C and SCA6

| **Required time** | **Group**  **difference** | **1st trial**  **vs. 2nd trial** | **2nd trial**  **vs. 3rd trial** | **1st trial**  **vs. 3rd trial** |
| --- | --- | --- | --- | --- |
|  | *p*-value | *p*-value | *p*-value | *p*-value |
| Patients with MSA-C | < 0.001* | < 0.001* | 0.057 | < 0.001* |
| Patients with SCA6 | 0.122 | NS | NS | NS |

| **Movement speed** | **Group**  **difference** | **1st trial**  **vs. 2nd trial** | **2nd trial**  **vs. 3rd trial** | **1st trial**  **vs. 3rd trial** |
| --- | --- | --- | --- | --- |
|  | *p*-value | *p*-value | *p*-value | *p*-value |
| Patients with MSA-C | 0.060 | NS | NS | NS |
| Patients with SCA6 | 0.497 | NS | NS | NS |

Abbreviations: MSA-C, multiple system atrophy with predominant cerebellar ataxia; SCA6, spinocerebellar ataxia type 6; NS, not significant
